# Standardised exhaled breath collection for the measurement of exhaled volatile organic compounds by proton transfer reaction mass spectrometry

**DOI:** 10.1186/1471-2466-13-43

**Published:** 2013-07-09

**Authors:** Andras Bikov, Koralia Paschalaki, Ron Logan-Sinclair, Ildiko Horváth, Sergei A Kharitonov, Peter J Barnes, Omar S Usmani, Paolo Paredi

**Affiliations:** 1 Airway Disease Section, National Heart and Lung Institute, Imperial College, Dovehouse Street, London SW3 6LY, UK; 2 Department of Pulmonology, Semmelweis University, Budapest, Hungary

## Abstract

**Background:**

Exhaled breath volatile organic compound (VOC) analysis for airway disease monitoring is promising. However, contrary to nitric oxide the method for exhaled breath collection has not yet been standardized and the effects of expiratory flow and breath-hold have not been sufficiently studied. These manoeuvres may also reveal the origin of exhaled compounds.

**Methods:**

15 healthy volunteers (34 ± 7 years) participated in the study. Subjects inhaled through their nose and exhaled immediately at two different flows (5 L/min and 10 L/min) into methylated polyethylene bags. In addition, the effect of a 20 s breath-hold following inhalation to total lung capacity was studied. The samples were analyzed for ethanol and acetone levels immediately using proton-transfer-reaction mass-spectrometer (PTR-MS, Logan Research, UK).

**Results:**

Ethanol levels were negatively affected by expiratory flow rate (232.70 ± 33.50 ppb vs. 202.30 ± 27.28 ppb at 5 L/min and 10 L/min, respectively, p < 0.05), but remained unchanged following the breath hold (242.50 ± 34.53 vs. 237.90 ± 35.86 ppb, without and with breath hold, respectively, p = 0.11). On the contrary, acetone levels were increased following breath hold (1.50 ± 0.18 ppm) compared to the baseline levels (1.38 ± 0.15 ppm), but were not affected by expiratory flow (1.40 ± 0.14 ppm vs. 1.49 ± 0.14 ppm, 5 L/min vs. 10 L/min, respectively, p = 0.14). The diet had no significant effects on the gasses levels which showed good inter and intra session reproducibility.

**Conclusions:**

Exhalation parameters such as expiratory flow and breath-hold may affect VOC levels significantly; therefore standardisation of exhaled VOC measurements is mandatory. Our preliminary results suggest a different origin in the respiratory tract for these two gasses.

## Background

There is a need for disease biomarkers that reflect the activity of the underlying pathogenetic pathways that characterise lung disease. These could help diagnose and monitor lung disorders besides providing information on the efficacy of treatment.

In the last decades breath analysis, and particularly the measurement of exhaled nitric oxide (NO), has received a lot of interest because its measurement is simple and its breath levels reflects airway inflammation [1]. Volatile organic compounds (VOCs) have also been shown to be elevated in inflammatory diseases [2-5], and also to reflect the activity of specific metabolic pathways. For example, acetone is linked to dextrose metabolism and lipolysis [5,6], whereas exhaled isoprene correlates with cholesterol biosynthesis [7], and exhaled levels of sulphur-containing compounds are elevated in liver failure [5,8] and allograft rejection [9]. Different VOC profiles have been identified in several diseases, such as lung cancer [10-13], asthma [14] and COPD [15] compared to controls, and the measurement of VOCs has been suggested as a tool for early detection and monitoring of disease.

Contrary to the measurement of exhaled NO, which has been carefully standardised, the parameters potentially affecting VOCs levels in the breath have received little notice [16,17]. The lack of standardization of the previously published methods and the poor knowledge of the variables that may affect VOCs have hindered the use of these gases in research. As a result, even though back in 1971 Pauling et. al. [18] detected more than 200 VOCs in the human breath, to date, breath analysis is still an underused research tool with no current clinical application.

In view of the potential usefulness of VOCs as markers of lung disease we developed a simple method for their measurement using Proton Transfer Reaction Mass Spectrometry (PTR-MS) and crucially, we standardised the breath collection and studied the effect of different breath parameters such as exhalation flow and breath hold on the levels of the measured gases.

Ethanol and acetone were chosen as test gases for standardization because of their ease of measurement and low concentrations in the environment.

## Methods

### Subjects and study design

Using the technique described above, exhaled breath was collected from 15 healthy non-smoking volunteers (mean age ± SEM, 34 ±7 yr; 9 males). Informed consent was obtained from all individuals. All subjects attended the Asthma Laboratory at the Royal Brompton Hospital on two occasions (visit 1 and visit 2) to verify the reproducibility of the measurements. None of the participating subjects had respiratory tract infection in the 4 weeks preceding the study. Subjects were asked to abstain from food for at least 2 hours prior to each visit. During each visit exhaled VOCs were measured in the morning and a few hours apart in the afternoon. The study was approved by the Royal Brompton Hospital research ethics committee (08/H0709/2).

### Standardised exhaled air collection

All breath samples were collected using a standardised technique during a pressure and flow-controlled exhalation into a polyethylene reservoir as previously described [19]. Inhalation was performed through the nose, without pauses, from residual volume (RV) to total lung capacity (TLC) and was immediately followed by exhalation without breath-hold. Subjects aimed at a constant exhalation flow rate (5 to 6 L/min) using a visual feedback. A resistance of 5 cmH_2_O was implemented in order to increase the mouth pressure and close the soft palate reducing the contamination of exhaled breath with nasal air [20].

The air coming from the dead space, contaminated with nasal and ambient air, was discarded in the atmosphere, by a three way valve [19]. The time needed to wash out the dead space (t) was estimated to be 1–2 seconds (s) (t = dead space volume/exhalation flow where dead space is calculated as weight (lb) + age in years, and exhalation flow is 5–6 L/min) [21], and therefore discard of the first 3 seconds of exhaled air could secure the removal of the dead space. At the end of the exhalation manoeuvre, the three way valve was promptly closed to avoid ambient contamination. Samples of ambient air were also collected at the same time.

### Sample analysis by PTR-MS

Breath samples and samples of ambient air were analysed by a proton transfer reaction mass spectrometer (PTR-MS) (Logan Research Ltd, Rochester, UK). Proton-transfer-reaction mass spectrometry was used to measure concentrations of VOCs in human breath at levels of ppb or even ppt as described by Hansel et al. 1995 [22]. Briefly, PTR-MS uses a soft ionization method based on proton transfer from H_3_O^+^ ions to all compounds with a higher proton affinity than water:

$$H_{3}O^{+}+R\to {\mathrm{RH}}^{+}+H_{2}O^{+},$$

where R is the reactant gas added, able to react with H_3_O^+^. The common constituents of air such as N_2_, O_2_, Ar, CO_2_ etc. have lower proton affinity than water and are therefore not detected. The reaction product ions are mass analysed using a quadrupole mass spectrometer and detected by a secondary electron multiplier (SEM). The ion detection system measures count rates i(H_3_O^+^) and i(RH^+^), which are proportional to the respective densities of these ions [23].

The system can reach higher sensitivity by not diluting the gas to be analysed in an additional buffer gas [23]. For the measurement of ethanol (atomic mass unit (amu) 47) the PTR-MS analyser was set at precision 4 and sensitivity 13 while acetone (59 amu) was detected at sensitivity 12 and precision 4. Each sample (breath sample or sample of ambient air) was analysed three consecutive times with two different sensitivities (sensitivity 12 and 13).

### Parameters affecting VOCs levels

#### *Breathing parameters*

In order to investigate and standardise the effect of different breathing parameters on VOCs levels, breath samples were collected using the technique described above but modifying the following:

a) 20 seconds breath hold

b) inclusion of dead space in the breath sample

c) exhalation flow rate of 5–6 L/min vs.10-11 L/min

In addition, we also studied the concentration of exhaled VOCs in the reservoir over 48 h.

#### *Diet*

Breath samples were collected in a subgroup of five healthy non-smoker volunteers (mean age +/- SEM, 38 +/- 4 yr; 3 males) after overnight fasting at 30, 120 and 210 minutes following a set breakfast (250 ml chocolate milk, 1 chocolate croissant) and at 30 and 150 minutes following a set lunch (chicken Caesar salad wrap, crisps) meal without any alcohol consumption.

#### *Alcohol*

Exhaled ethanol was measured in four healthy non-smoker volunteers (mean age +/- SEM, 35 +/- 3 yr; 3 males) after at least 3 hours fasting and at 5 minutes, 1 hour, 2.5, 3.5 and 4.5 hours following the consumption of 1.5 units of alcohol (125 ml of wine, 12%).

#### *Effect of ambient air*

The levels of ethanol and acetone were measured in breath samples and a correlation with the concentration of the same gases in the environmental air was investigated.

### Inter-session and intra-session reproducibility

VOCs measurements were carried out twice on the same day at least two hours apart (visit one, inter-session reproducibility) and 3–4 days later (visit one vs. visit 2, intra-session reproducibility).

#### *Sample statistical analysis*

The effects of exhalation flow rate, breath hold and dead space were analysed using the paired t-tests. The reproducibility of the method was assessed by Bland Altman test. The correlation of breath samples with samples of ambient air was estimated with the Pearson test. Significance was defined as a p-value of < 0.05. GraphPad Prism statistical package was used.

## Results

### Parameters affecting VOCs levels

#### *Exhalation flow*

**Figure 1:**
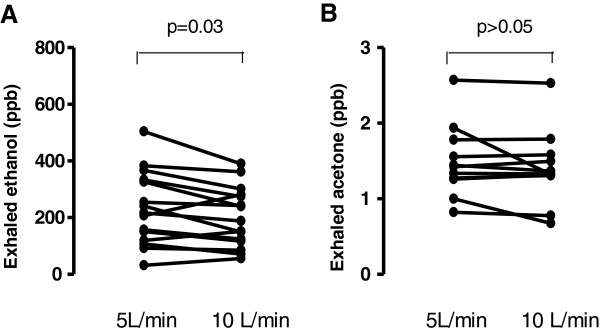
Exhaled ethanol (Panel A) and acetone (Panel B) levels at exhalation flow rates of 5 and 10 L/min.

Exhaled ethanol levels were significantly lower at an exhalation flow of 10 L/min (202.30 ± 27.28 ppb) compared to 5 L/min (232.70 ± 33.50 ppb, p = 0.03, Figure 1, Panel A) whereas the concentrations of acetone were not affected by different exhalation flow rates (1.40 ± 0.14 ppm, 1.49 ± 0.14 ppm for 5 and 10 L/min exhalations respectively, Figure 1, Panel B).

#### *Breath hold*

**Figure 2:**
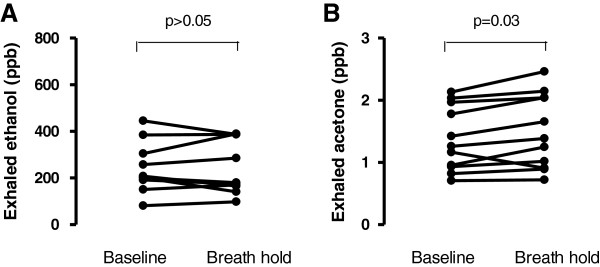
Exhaled ethanol (Panel A) and acetone (Panel B) levels at baseline and following a 20 secods breath hold.

Exhaled ethanol levels were unchanged before (242.50 ± 34.53 ppb) and after a 20 second breath-hold manoeuvre (237.90 ± 35.86 ppb, (p > 0.05)) (Figure 2, panel A). However, acetone levels were significantly affected (1.38 ± 0.15 ppm, 1.50 ± 0.18 ppm at baseline and after breath hold respectively, p = 0.03, Figure 2 Panel B).

#### *Dead space*

**Figure 3:**
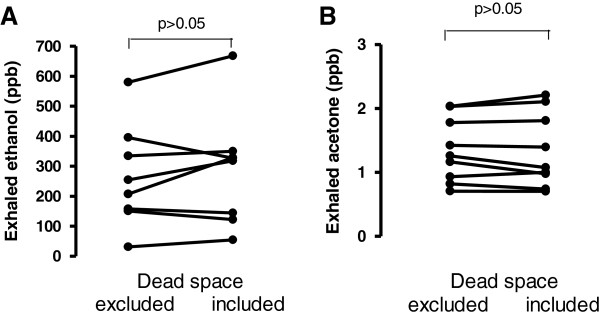
Exhaled ethanol (Panel A) and acetone (Panel B) levels with ant without the exclusion of dead space air.

The inclusion of exhaled dead space air in the analysed samples did not affect ethanol (264.00 ± 60.37 ppb and 289.00 ± 67.47 ppb with and without dead space air respectively, p > 0.05, Figure 3, Panel A) or acetone levels (1.35 ± 0.16 ppm and 1.33 ± 0.19 ppm with or without dead space respectively, p > 0.05, Figure 3, Panel B).

#### *Diet and alcohol*

**Figure 4:**
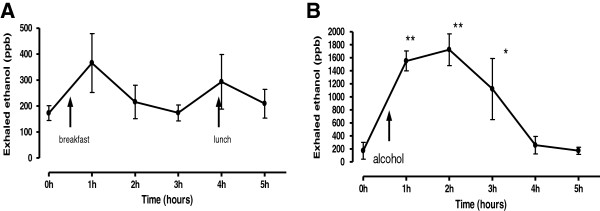
Exhaled ethanol levels following breakfast, lunch (Panel A) and alcohol consumption (Panel B). ** = p < 0.01, * = P < 0.05.

There was a tendency for higher ethanol levels 30 min after both breakfast and lunch compared to the overnight fasting concentrations (Figure 4, Panel A) however, these changes were not statistically significant.

As expected, there was a very significant and rapid increase of exhaled ethanol concentrations 5 minutes after wine consumption, these levels remained significantly increased for 2.5 hours and returned to baseline levels after 3.5 hours (Figure 4, Panel B).

#### *Effect of ambient air*

In order to investigate the influence of environmental air on the levels of VOCs in the breath, we correlated the levels of VOCs in breath samples with those measured in environmental samples. Over a large range of concentrations of a number of measured VOCs (ethanol, methanol, isoprene, acetonitrile, phenol) using two different sensitivities used (12 and 13), we were unable to find any significant correlations.

#### *Reproducibility*

**Figure 5:**
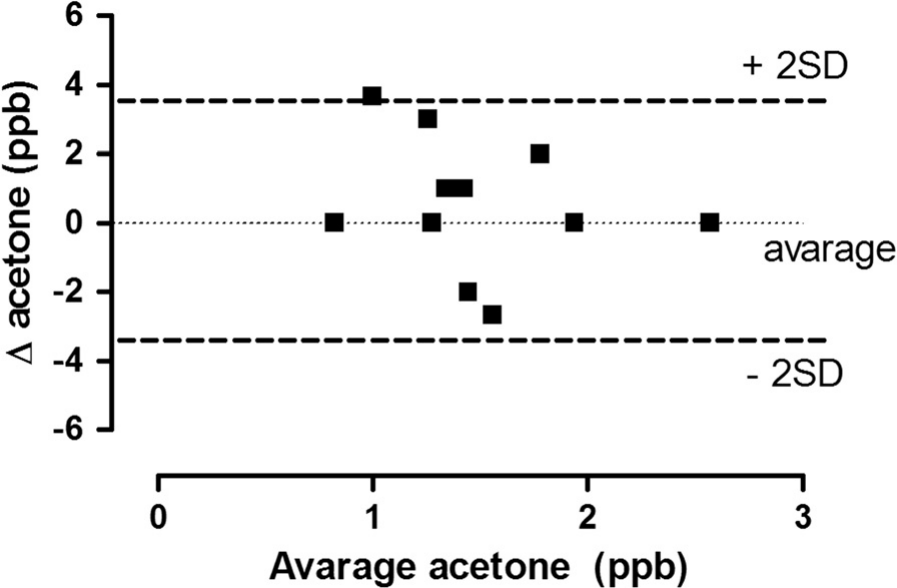
**Repeatability of acetone measurement.** Two exhaled acetone measurements separated by five minute intervals in 15 normal volunteers.

The difference in exhaled, ethanol, and acetone levels measured during two collections made the same day (comparison between session in the same visits) plotted against their mean (Bland and Altman test, single session variability) showed that most of the measurements were within 2SD of the mean (Figure 5). The inter session variability (comparison between different visits days apart) also satisfied the Bland and Altman test. The coefficients of variation for ethanol and acetone were 2.41% and 13.9% respectively.

Even though breath samples were analysed immediately after collection, the concentration of ethanol and acetone in five breath collection reservoir was stable over the course of 48 h.

## Discussion

We standardised breath sample collection and developed a new method for VOC analysis. Because exhalation flow rate and breath hold may affect the expired levels of ethanol and acetone, we suggest that controlling breathing parameters is required to reduce errors and improve the reproducibility of VOC measurements.

Contrary to the measurement of exhaled NO which has been extensively investigated and standardised as described in joint ERS/ATS guidelines [24], only two preliminary studies have so far investigated the breathing parameters potentially affecting the levels of VOCs in the exhaled breath [16,17]. Notably, none of the so far published clinical studies controlled or investigated the effect of breathing parameters on VOC levels. In the current manuscript, we used a previously developed device for exhaled breath collection which allowed us to analyse separately the effect of different breathing manoeuvres.

Ethanol breath levels were significantly decreased at higher exhalation flow rates. As the central airway axial diffusion is an important factor determining flow dependency [25] this may indicate that ethanol has a significant axial diffusion and the central airways contribute significantly to total ethanol breath levels. Three subjects with low baseline ethanol levels showed no exhalation flow dependency, suggesting that higher gas levels may be more sensitive flow rate reduction. The flow rate of 5 L/min may be a more suitable standard as it is more comfortable. Interestingly, contrary to ethanol, acetone breath concentrations were not exhalation flow rate dependent indicating a poor contribution of the central airways to the total acetone concentrations in the breath. Alternatively, the lack of exhalation flow dependency may result from back diffusion into the tissues allowing the gases to be washed away by the blood stream before axial diffusion can occur. The lack of exhalation flow dependency of acetone shown in a previous study [16] may be related to the use of much higher exhalation flow rates (15 l/min) which may have cancelled the effect of this variable. The significant effect produced by breath hold on the levels of acetone supports the theory that this gas may have an elevated airway uptake as opposed to ethanol which was not significantly affected by breath hold and therefore may have a higher central airway production/diffusion ration as suggested by its significant flow dependency and lack of reuptake.

Dead space air is mostly a mixture of nasal and ambient air. It is reassuring that the inclusion of dead space air in the breath analysis did not affect the final VOC levels as this suggests low upper airways and nasal VOC concentrations as well as low environmental levels of the measured gases. Even though dead space air did not affect the level of the gasses measured, we advise discarding its collection to reduce the possibility of sporadically elevated environmental levels of acetone and ethanol.

The breathing parameters studied may affect exhaled gases differently depending on their biological and physical properties, therefore, it is crucial to underline that other gases not measured in the current study, with different biophysical properties may be affected. Therefore, we suggest that exhaled breath for VOC analysis should always be collected in a standardised manner as described in the current manuscript.

The presence of water vapour in the exhaled breath presents a technical challenge as it interferes with the measurement of other gases with molecular weights close to that of water. In order to reduce this error, we have limited our analysis to gases with molecular weight dissimilar from water. This approach together with a controlled collection of the exhaled breath has provided an excellent inter and intra- session reproducibility.

Because VOCs are present in the exhaled breath at very low concentrations, another potential error may derive from contamination with environmental air. Some authors have minimised this problem by concentrating the exhaled breath [26-28] or passing it through a scrubber [29,30]. Notably, the lack of a significant correlation between exhaled breath VOCs and the concentration of the same gases in concurrent ambient samples may indicate that environmental contamination was not relevant in our study. We controlled environmental contamination by reducing air leaks in the tubing system and carefully sealing the reservoir where the exhaled breath was collected.. Furthermore, exhaling against a resistance producing a mouth pressure of at least 5 cm H_2_O may have reduced contamination of the exhaled breath with nasal and environmental air by closing the soft palate as previously described [20].

Other factors which potentially influence the levels of VOCs in the breath are diet [31-34] and alcohol consumption [35]. As expected, alcohol rapidly and significantly increased the levels of exhaled ethanol which gradually decreased and returned to baseline levels 3.5 hours after wine consumption. This supports the hypothesis that breath ethanol levels reflect a metabolic process and not alcohol vapours coming from the stomach immediately after drinking wine.

Previous reports have shown that the diet may affect the levels of ethanol [36,37]. In our study there was a tendency for higher levels of breath ethanol 30 minutes after the ingestion of food however this was not statistically significant. Interestingly, there was a trend for increased ethanol levels following breakfast rather than lunch even though none of them was statistically significant. This may be related to the higher content of carbohydrates in the former which may have been metabolised to form ethanol.

## Conclusions

We analysed the factors potentially affecting the levels of some VOCs in the breath and standardised a method for their measurement controlling breathing parameters and diet. Under these conditions, we demonstrated that both intra-session and inter-session reproducibility of the method were satisfactory. We believe that the standardization of the method for the measurement of VOCs in the breath is necessary to provide the reliability required for its research and clinical use and we suggest that the study of new VOCs should include a thorough analysis of their physiology as this may provide information on the origin of any specific VOC in the respiratory tract.

## Abbreviations

ATS: American thoracic society; COPD: Chronic obstructive pulmonary disease; FeNO: Fraction of exhaled nitric oxide; FEV1: Forced expiratory volume in one second; PTR-MS: Proton transfer reaction mass spectrometry; RV: Residual volume; TLC: Total lung capacity; VOC: Volatile organic compounds.

## Competing interests

I confirm that I have no competing interests. I did not receive any fees or funding or have stocks or shares in organizations that may benefit from the publishing of this paper. I am not applying for patents related to the content of this manuscript. I do not have any non-financial competing interests.

## Authors’ contributions

AB and KP equally contributed to the manuscript. They both participated in developing the concept and design of the study. In addition, they collected and analysed the data and edited the final draft of the manuscript. RLS built the VOC analyser used in this study, he also participated in the design of the study and writing and editing of the final draft. IH, SAK and PJB participated in the development of the concept, study design, writing and editing of the final draft. PP had a major role designing the study, he also participated in patients recruitment, helped data analysis and supervised the progress of the study, in addition, he wrote the first draft of the manuscript and re edited it following all the other authors input. All authors read and approved the manuscript.

## Pre-publication history

The pre-publication history for this paper can be accessed here:

http://www.biomedcentral.com/1471-2466/13/43/prepub

## References

[B1] Barnes PJ Dweik RA Gelb AF Gibson PG George SC Grasemann H Exhaled nitric oxide in pulmonary diseases: a comprehensive review Chest 2010 138 3 682 692 10.1378/chest.09-2090 20822990

[B2] Paredi P Kharitonov SA Loukides S Pantelidis P du Bois RM Barnes PJ Exhaled nitric oxide is increased in active fibrosing alveolitis Chest 1999 115 5 1352 1356 10.1378/chest.115.5.1352 10334152

[B3] Paredi P Kharitonov SA Barnes PJ Elevation of exhaled ethane concentration in asthma Am J Respir Crit Care Med 2000 162 4 Pt 1 1450 1454 11029360 10.1164/ajrccm.162.4.2003064

[B4] Paredi P Kharitonov SA Leak D Ward S Cramer D Barnes PJ Exhaled ethane, a marker of lipid peroxidation, is elevated in chronic obstructive pulmonary disease Am J Respir Crit Care Med 2000 162 2 Pt 1 369 373 10934055 10.1164/ajrccm.162.2.9909025

[B5] Miekisch W Schubert JK Noeldge-Schomburg GF Diagnostic potential of breath analysis–focus on volatile organic compounds Clin Chim Acta 2004 347 1–2 25 39 15313139 10.1016/j.cccn.2004.04.023

[B6] Crofford OB Mallard RE Winton RE Rogers NL Jackson JC Keller U Acetone in breath and blood Trans Am Clin Climatol Assoc 1977 88 128 139 898531 PMC2441399

[B7] Stone BG Besse TJ Duane WC Evans CD DeMaster EG Effect of regulating cholesterol biosynthesis on breath isoprene excretion in men Lipids 1993 28 8 705 708 10.1007/BF02535990 8377584

[B8] Chen S Zieve L Mahadevan V Mercaptans and dimethyl sulfide in the breath of patients with cirrhosis of the liver. Effect of feeding methionine J Lab Clin Med 1970 75 4 628 635 5444348

[B9] Studer SM Orens JB Rosas I Krishnan JA Cope KA Yang S Patterns and significance of exhaled-breath biomarkers in lung transplant recipients with acute allograft rejection J Heart Lung Transplant 2001 20 11 1158 1166 10.1016/S1053-2498(01)00343-6 11704475

[B10] Horvath I Lazar Z Gyulai N Kollai M Losonczy G Exhaled biomarkers in lung cancer Eur Respir J 2009 34 1 261 275 10.1183/09031936.00142508 19567608

[B11] Westhoff M Litterst P Freitag L Urfer W Bader S Baumbach JI Ion mobility spectrometry for the detection of volatile organic compounds in exhaled breath of patients with lung cancer: results of a pilot study Thorax 2009 64 9 744 748 10.1136/thx.2008.099465 19158121

[B12] Bajtarevic A Ager C Pienz M Klieber M Schwarz K Ligor M Noninvasive detection of lung cancer by analysis of exhaled breath BMC Cancer 2009 9 348 10.1186/1471-2407-9-348 19788722 PMC2761408

[B13] Poli D Goldoni M Corradi M Acampa O Carbognani P Internullo E Determination of aldehydes in exhaled breath of patients with lung cancer by means of on-fiber-derivatisation SPME-GC/MS J Chromatogr B Analyt Technol Biomed Life Sci 2010 878 2643 2651 10.1016/j.jchromb.2010.01.022 20149763

[B14] Ibrahim B Basanta M Cadden P Singh D Douce D Woodcock A Non-invasive phenotyping using exhaled volatile organic compounds in asthma Thorax 2011 66 9 804 809 10.1136/thx.2010.156695 21749985

[B15] Fens N Zwinderman AH van der Schee MP de Nijs SB Dijkers E Roldaan AC Exhaled breath profiling enables discrimination of chronic obstructive pulmonary disease and asthma Am J Respir Crit Care Med 2009 180 11 1076 1082 10.1164/rccm.200906-0939OC 19713445

[B16] Boshier PR Priest OH Hanna GB Marczin N Influence of respiratory variables on the on-line detection of exhaled trace gases by PTR-MS Thorax 2011 66 10 919 920 10.1136/thx.2011.161208 21474496

[B17] Thekedar B Oeh U Szymczak W Hoeschen C Paretzke HG Influences of mixed expiratory sampling parameters on exhaled volatile organic compound concentrations J Breath Res 2011 5 1 016001 10.1088/1752-7155/5/1/016001 21383425

[B18] Pauling L Robinson AB Teranishi R Cary P Quantitative analysis of urine vapor and breath by gas–liquid partition chromatography Proc Natl Acad Sci U S A 1971 68 10 2374 2376 10.1073/pnas.68.10.2374 5289873 PMC389425

[B19] Paredi P Loukides S Ward S Cramer D Spicer M Kharitonov SA Exhalation flow and pressure-controlled reservoir collection of exhaled nitric oxide for remote and delayed analysis Thorax 1998 53 9 775 779 10.1136/thx.53.9.775 10319060 PMC1745326

[B20] Kharitonov SA Barnes PJ Nasal contribution to exhaled nitric oxide during exhalation against resistance or during breath holding Thorax 1997 52 6 540 544 10.1136/thx.52.6.540 9227721 PMC1758589

[B21] Cotes JE Lung function. Assessment and application in medicine 1994 Fifth edition [21] Oxford: Blackwell Scientific Publications

[B22] Hansel A Jordan A Holzinger R Prazeller P Vogel W Lindinger W Proton transfer reaction mass spectrometry: on-line trace gas analysisi at the ppb levels Int J Mass Spect Ion Proc 1995 149/150 609 619

[B23] Lindinger W Hansel A Jordan A Proton-transfer-reaction mass spectrometry (PTR-MS): on-line monitoring of volatile organic compounds at pptv levels Chem Soc Rev 1998 27 347 354 10.1039/a827347z

[B24] Recommendations for standardized procedures for the on-line and off-line measurement of exhaled lower respiratory nitric oxide and nasal nitric oxide in adults and children-1999. This official statement of the american thoracic society was adopted by the ATS board of directors Am J Respir Crit Care Med 1999 160 2104 2117 10588636 10.1164/ajrccm.160.6.ats8-99

[B25] Kerckx Y Van MA Axial distribution heterogeneity of nitric oxide airway production in healthy adults J Appl Physiol 2009 106 6 1832 1839 10.1152/japplphysiol.91614.2008 19342432

[B26] Amorim LC de L Cardeal Z Breath air analysis and its use as a biomarker in biological monitoring of occupational and environmental exposure to chemical agents J Chromatogr B Analyt Technol Biomed Life Sci 2007 853 1–2 1 9 17418649 10.1016/j.jchromb.2007.03.023

[B27] Sanchez JM Sacks RD GC analysis of human breath with a series-coupled column ensemble and a multibed sorption trap Anal Chem 2003 75 10 2231 2236 10.1021/ac020725g 12918960

[B28] Sanchez JM Sacks RD Development of a multibed sorption trap, comprehensive two-dimensional gas chromatography, and time-of-flight mass spectrometry system for the analysis of volatile organic compounds in human breath Anal Chem 2006 78 9 3046 3054 10.1021/ac060053k 16642992

[B29] Raymer JH Thomas KW Cooper SD Whitaker DA Pellizzari ED A device for sampling of human alveolar breath for the measurement of expired volatile organic compounds J Anal Toxicol 1990 14 6 337 344 10.1093/jat/14.6.337 2128356

[B30] Wallace L Buckley T Pellizzari E Gordon S Breath measurements as volatile organic compound biomarkers Env Health Persp 1996 104 Suppl 5 861 869 10.1289/ehp.96104s5861 PMC1469714 8933027

[B31] Lindinger W Taucher J Jordan A Hansel A Vogel W Endogenous production of methanol after the consumption of fruit Alcohol Clin Exp Res 1997 21 5 939 943 10.1111/j.1530-0277.1997.tb03862.x 9267548

[B32] Smith D Spanel P Davies S Trace gases in breath of healthy volunteers when fasting and after a protein-calorie meal: a preliminary study J Appl Physiol 1999 87 5 1584 1588 10562594 10.1152/jappl.1999.87.5.1584

[B33] Turner C Spanel P Smith D A longitudinal study of methanol in the exhaled breath of 30 healthy volunteers using selected ion flow tube mass spectrometry, SIFT-MS Physiol Meas 2006 27 7 637 648 10.1088/0967-3334/27/7/007 16705261

[B34] Turner C Spanel P Smith D A longitudinal study of ammonia, acetone and propanol in the exhaled breath of 30 subjects using selected ion flow tube mass spectrometry, SIFT-MS Physiol Meas 2006 27 4 321 337 10.1088/0967-3334/27/4/001 16537976

[B35] Smith D Pysanenko A Spanel P Kinetics of ethanol decay in mouth- and nose-exhaled breath measured on-line by selected ion flow tube mass spectrometry following varying doses of alcohol Rapid Commun Mass Spect 2010 24 7 1066 1074 10.1002/rcm.4481 20213689

[B36] Turner C Spanel P Smith D A longitudinal study of ethanol and acetaldehyde in the exhaled breath of healthy volunteers using selected-ion flow-tube mass spectrometry Rapid Commun Mass Spect 2006 20 1 61 68 10.1002/rcm.2275 16312013

[B37] Kharitonov SA Religious and spiritual biomarkers in both health and disease Religions 2012 3 2 467 497

